# The Effects of Workplace Nature-Based Interventions on the Mental Health and Well-Being of Employees: A Systematic Review

**DOI:** 10.3389/fpsyt.2020.00323

**Published:** 2020-04-28

**Authors:** Susan Gritzka, Tadhg E. MacIntyre, Denise Dörfel, Jordan L. Baker-Blanc, Giovanna Calogiuri

**Affiliations:** ^1^Health Research Institute, University of Limerick, Limerick, Ireland; ^2^School of Science, Faculty of Psychology, Technische Universität Dresden, Dresden, Germany; ^3^Department of Physical Education and Sport Sciences, University of Limerick, Limerick, Ireland; ^4^Department of Public Health and Sport Sciences, Faculty of Social and Health Sciences, Inland Norway University of Applied Sciences, Elverum, Norway

**Keywords:** employees, environmental psychology, health promotion, green exercise, mental health, occupational health, occupational psychology

## Abstract

Mental health in the workplace is a societal challenge with serious economical and human costs. Most prevalent mental disorders in the workforce (e.g., depression), however, are preventable. There is widespread agreement about the favorable effects of nature exposure and consequently, nature-based interventions (NBI) in the workplace have been proposed as a cost-effective approach to promote good health among employees. The objective of the present study was to systematically review scientific evidence on the effectiveness of NBI to promote mental health and well-being among actual employees in actual workplace settings. The review was conducted and presented in accordance with the PRISMA guidelines. The literature search was performed on five databases (PubMed, Embase, CENTRAL, CINHAL, and PsycINFO), hand-searching of field-specific journals, and the reference lists of retrieved papers over the past 5 years up to November (13^th^, 2018). Studies were eligible for inclusion if they (*i)* were randomized or nonrandomized controlled trials; (*ii)* comprised samples of actual employees; (*iii)* implemented a workplace-based intervention with exposure to nature; (*iv)* included comparison conditions that displayed a clear contrast to NBIs; and (*v)* investigated the quantitative effects on mental health or well-being. No restrictions on type of employees or workplace, publication period, or language of the publication were set. Risk of bias was assessed using the Cochrane’s RoB2 tool. Narrative synthesis was performed due to large heterogeneity in outcome variables. Of the 510 articles identified, 10 NBIs (nine papers) met the eligibility criteria. The outcomes were grouped in five categories: (*i)* mental health indices, (*ii)* cognitive ability, (*iii)* recovery and restoration, (*iv)* work and life satisfaction, and (*v)* psychophysiological indicators. Narrative synthesis indicates consistently positive effects on mental health indices and cognitive ability, while mixed results were found for the other outcome categories. Caution must be given when interpreting the current evidence in this emerging research field because of the diversity of NBIs and the overall high risk of bias in the individual studies. Although in this field often researchers have to balance scientific rigor and ecological validity, there is a need for large, well-designed and rigorously conducted trials grounded in contemporary theories.

## Introduction

It is time to expand the remit of occupational health psychology (1) due to the complexity (2), change (3), and globalization (4) of the labor market. A recent meta-analysis determined that workplace factors including imbalanced job design, occupational uncertainty, and lack of value and respect in the workplace, contribute to poor mental health (5). Emerging trends in occupational health psychology demonstrate a paradigm shift, an advocated turn toward positive psychology, which, instead of addressing mental illness and risk factors aims to focus on fostering employees’ mental health, well-being, and cultivating a healthy workplace (6, 7). In spite of the evidence supporting the effectiveness of positive psychology interventions in the workplace (8), and although working life often involves the need to recover from stress, many workplaces are, in practice, often neglected as a setting for implementing positive preventive approaches. Yet, the working environment represents a vital and ideal context for the promotion of mental health (1, 9) which is an important complement to clinical mental health interventions (10).

### Nature Exposure and Nature-Based Interventions

There is a general consensus about the favorable effects of nature exposure (e.g., viewing or spending time in green and blue space) within several systematic and narrative reviews [e.g., (11–18)]. A recent systematic review of 12 reviews underlined the benefits of exposure to nature in all-cause mortality, mortality by cardiovascular diseases, and mental health among adult populations (19). However, uncertainty about context-specific evidence (e.g., work setting) remains. Two main theories, attention restoration theory [ART; (20–22)], and stress reduction theory [SRT; (23–25)] outline a critical role for nature contact in terms of health. ART [e.g., (26)] holds a cognitive explanation as a prolonged focus on demanding (work) activity leads to mental fatigue and further to negative emotional states (e.g., lack of energy) as well as to impairments in cognitive and physical performance. Particularly, according with ART, natural stimuli attract spontaneous interest and enable restoration, i.e. renewal of depleted resources (e.g., capacity of directed attention) (27). Restoration refers to feeling refreshed, attentionally recovered coupled with positive emotions, and low levels of stress and arousal (28). SRT [e.g., (25)] elucidates the restorative impacts of nature on effective functioning (i.e. eudaimonic well-being) and emotional well-being (i.e. hedonic well-being). According to the theory, as a result of evolutionary development, individuals have an innate predisposition to automatically and immediately exhibit positive affect toward natural, vegetation-rich environments resulting in stress-reducing psychophysiological responses (29, 30).

Both, the evidence and the theoretical underpinning of the health benefits of nature exposure, lay the ground for so-called nature-based interventions (NBI). A generally accepted definition of NBI is lacking (31). Moreover, numerous terms are used such as nature-assisted interventions (32), nature-based therapeutic interventions (33), green care (34), and ecotherapy (35). In this review, based on revising previous operational definitions [e.g., (36)], we define NBIs as planned, intentional activities to promote individuals’ optimal functioning, health and well-being or to enable restoration and recovery through exposure to or interaction with either authentic or technological nature. We augment other NBIs definition by including technological nature [e.g., through virtual computer-generated nature settings; (37, 38)] and by encompassing “recovery” in order to account for work and organizational psychology constructs, too.

### Mechanisms Linking NBIs to Health and Well-Being

Both, ART (20) and SRT (25) discuss restoration as a core process, which is assumed to be triggered by spending time in nature. Restoration hereby is related to cognitive recovery, positive emotions and hedonic well-being, as well as low levels of stress and arousal (28). Focusing on the work context, the *Job Demands-Resources Model* [JD-R; (39)] predicts that personal resources can buffer the negative effects of (adverse) job demands on well-being (e.g., burnout). If those personal resources are depleted, they have to be restored, which can be achieved by certain activities (40, 41). Those activities are supposed to have certain characteristics, for instance psychological detachment and relaxation (41), which are very similar to the characteristic effects of nature exposure as proposed by ART and SRT. Nature based interventions trigger psychological detachment (mental disengagement from work, attentional recovery) because according to evolutionary perspectives humans are predisposed to pay attention to natural environments (see e.g., ART, as described in the previous paragraph). Work-related stress exposure is reduced, attention is directed away from job demands toward natural stimuli, which might allow the renewal of (work related) attentional capacity [see (27, 42)]. Additionally, SRT implies that nature contact elicits positive affect (because places rich in water and vegetation were favorable to survival or ongoing well-being). Positive affect in turn is influencing physiological stress responses (either acute or chronic), which in turn prepares the organism for appropriate adaptive behaviors (43). The specific environmental conditions provided by nature might offer an explanation for the favorable effects on physiological stress responses [see (44)], and therefore the support of relaxation. Plants, for instance, emit *phytoncides*, which have been shown to reduce blood pressure and alter autonomic activity. Other mechanisms that have been investigated are environmental biodiversity, negative air ions, microorganisms, less air pollution, cooler temperatures *via* their effects on cardiovascular, autonomic, gastro-intestinal and immune functioning, anti-obesity, and anti-diabetic processes (42, 44). A detailed presentation of the mechanisms, however, is beyond the scope of this review.

### A Positive Occupational Health Psychology Perspective on NBIs

In this perspective, NBIs also stem from a salutogenic approach (33, 45) and can be classified as positive occupational health psychology interventions (POHP), which aim to support optimal functioning of people, groups, and organizations (46, 47). Workplace interventions can be categorized as primary, secondary, and tertiary. Primary workplace prevention interventions seek to counter the incidence of mental health issues by changing the work environment (48). From a primary prevention perspective, POHP emphasize the importance for an organizational approach centralizing the advancement of resources, development of strengths (49, 50), and the cultivation of subjective well-being and mental health (51). Workplace interventions at the secondary level are “ameliorative and worker-directed,” aiming to modify employees reaction, coping and resilience toward stress, thereby preventing the progression of subclinical mental health symptoms to diagnosable conditions [(51), p. 3]. In terms of treating mental illnesses, tertiary prevention interventions aim to minimize its impairment on a person’s functioning (52). NBIs can be allocated to either of those, depending on their application and implementation as NBIs comprise a high diversity in their design, settings, target populations (53, 54), and goals (55).

Particularly, the POHP view of mental health in the workplace as on a continuum, varying from flourishing to languishing (56, 57) is useful to the understanding of how NBIs can promote health and well-being to employees. Languishing individuals perceive their work and life as “hollow” or “empty” (58) and it can occur with or without the presence of a diagnosed mental illness. Yet, both states are dysfunctional and translate to reduced levels of well-being. Hence, the critical question is how to foster flourishing in the workplace. In organizational psychology the JD-R model (59, 60) describes workplaces as a function of job demands (e.g., work pressure), job resources (e.g., social support), and personal resources (e.g., self-efficacy). According to this model, strain arises when job demands exceed the employees’ belief in their capability to cope with them. Further, the depletion or lack of personal resources increases the risk of poor mental health. Recovery enables employees to restore their resources in order to preserve full working capacities and physical and mental health (40). Exposure to nature can help employees to fulfill all four recovery experiences and thereby enable psychophysiological unwinding. According to Sonnentag and Fritz (41) an activity needs to be characterized by four specific recovery experiences to ensure recovery: (*i)* psychological detachment (i.e. disengaging mentally from work); (*ii)* relaxation (i.e. low sympathetic activation plus positive affect); (*iii)* mastery (i.e. experiencing competence and proficiency in nonwork related domains) and (*iv)* control (i.e. ability to choose and to decide which activity to pursue). NBI might provide all these experiences.

### An Integrative Theoretical Framework for NBIs

It should be noted that a large variety of NBI types exists (32), e.g., horticultural therapy (61), care farming (62), green exercise (63), wilderness therapy (64), and green exercise, the latter defined as “adopting physical activities while at the same time being directly exposed to nature” [(65), p. 6]. This variety implies the need of a broad and flexible theoretical framework that can be adapted, depending on type of NBI, to specific contexts.

The synthesis of theoretical accounts from environmental psychology [*ART*, (20); *SRT*, (25)], work and organizational psychology [*JD-R model*, 39; *COR*, e.g., (66)] and positive psychology [*Broaden-and-Build Theory of Positive Emotions*, (67)] builds a strong foundation for considering NBIs as an affordable, upstream workplace intervention. In particular, NBIs as a POHP intervention have the ability to increase positive emotions at the workplace and thereby offer a pathway toward optimal functioning and well-being for employees in the long term. POHP bridges the gap of solely focusing on curing mental illness by including dedicated, proactive, good mental health strategies (68). Overall, the implementation of NBIs is desirable from the perspective of employees, employers and society as a whole [e.g., (69)].

### State of the Art on NBIs and Purpose of the Present Study

In a systematic review, Annerstedt and Währborg (55) found consistent evidence for the effectiveness and appropriateness of NBIs as a novel approach in public health for varied states of ill health (e.g., mental and attentional fatigue, symptoms of depression, and mood disturbances). The systematic review revealed effects on psychological, social, and physical outcomes. A systematic review reported positive effects (i.e. greater feelings of revitalization, positive engagement, energy, reduced tension, confusion, anger, and depression) after only one single bout of green exercise as opposed to indoors (70). However, a subsequent systematic review highlighted that the evidence on the additional benefits of green exercise, as compared with indoor exercise, is still broadly mixed (71). Both reviews emphasize how methodological limitations of green exercise studies might explain such inconsistencies.

Experimental designs are rather scarce in both organizational contexts (72) and nature-related research (73) due to high realization costs, difficulties to implement and many confounding variables outside of the investigator’s control (74). As a result, a large number of experimental studies have been conducted among students’ populations in simulated work settings [e.g., (75, 76)] instead of actual employees in real work settings. Thus, questions concerning the generalizability of the results (55), the dosage of nature (77) respectively the duration of contact with nature (78) causal pathways (44) and the cost-effectiveness as well as what features of nature might be more beneficial than others (54) still have to be clarified. On the other hand, studies on actual employees in real workplace settings do exist, although to the best of our knowledge, a review that synthesize such knowledge is still missing.

The objective of this review is to systematically synthesize and assess the existing empirical research on mental health and well-being outcomes on actual employees attending NBIs in their workplace. The focus of the review is on preventative approaches (primary intervention), while studies with employees suffering from diagnosed psychopathology (secondary and tertiary interventions) are omitted. In particular, the following research questions guided this systematic review:

1. What types of NBIs have been applied in real workplace setting?
2. What are the differential effects of different types of NBIs on employee mental health and well-being?

## Methods

### Study Design

This systematic literature review employed the Preferred Reporting Items for Systematic Reviews and Meta-Analyses (PRISMA) guidelines (79, 80). Five databases (PubMed, Embase, CENTRAL, CINHAL, and PsycINFO) were searched systematically using keywords derived from the analysis of key studies (SG) (See **Table 1**).

**Table 1:** Example search strategy for PubMed.

Search number	Search terms/Combination	Hits
#5	#4 AND (“adult”[MeSH Terms] OR “adolescent”[MeSH Terms])	80
#4	#1 AND #2 AND #3	211
#3	mental health OR well-being OR well being OR wellbeing OR restoration OR recovery OR psychological health OR psychological stress OR work stress OR job stress OR stress-related health OR Relaxation OR Ill health OR positive affect	793,866
#2	greenspace* OR green space* OR bluespace* OR blue space* OR greenery OR outdoor OR outdoors OR nature exposure OR nature contact OR nature sound OR natural environment* OR restorative environment* OR natural setting* OR park OR forest OR office landscaping OR nature-based OR garden	95,471
#1	workplace OR workplaces OR work place OR work places OR office OR offices OR occupation OR occupations OR employee OR employees OR worker OR workers OR staff OR personnel	483,400

The search was limited to title and abstract.

The search strategy was designed by SG and reviewed by TEM. The entire bibliographic search was conducted on the 8^th^–13^th^ November 2018. Supplementary approaches of contacting key authors and hand searching for papers within the last five years (2013–2018) finalized the search process (SG, JBB). Hand-searching was conducted on the 14^th^–17^th^ November 2018 in the following journals: BMC Public Health [Vol. 13–Vol. 18, keyword: “employee”], Journal of Environmental Psychology [Vol. 33–Vol. 59], Journal of Experimental Psychology: Applied [Vol. 19–Vol. 24 (3)], Journal of Occupational and Environmental Medicine [Vol. 55, issue 1–Vol. 60, issue 11], Journal of Occupational Health Psychology [Vol. 18–Vol. 23], Journal of Workplace Behavior Health” [Vol. 28–Vol. 33] and Scandinavian Journal of Work, Environment and Health [Vol. 39, issue 1–Vol. 5]. It was decided to search only for peer-published literature and to exclude grey literature (e.g., Open Grey Database) as it limits precise conclusion about quality (81).

### Eligibility Criteria

Eligibility criteria were defined using the PICOS-Framework [e.g., (82)]:

- Population: employees;
- Intervention: any type of NBIs;
- Control: control group required;
- Outcome: any measurements of mental health and well-being assessed using questionnaire;
- Study Design: exclusion of observational studies.

#### Participants/Population

The intervention had to be a workplace-based intervention, targeting people who perform their job within the organization. NBI-related activities could occur elsewhere but had to be implemented in and/or by their workplace (i.e. location of employment) or offered by the employers. Studies introducing a nonworkplace intervention (e.g., community intervention) including persons in employment were not eligible. With regards to employee populations, a specific inclusion criteria was that only adults (≥ 18 years) were included in the samples. Experimental studies that aimed to create a realistic office setting but did not reflect a real workplace with employees (e.g., student populations) were excluded. Moreover, employees that suffered from a diagnosed mental illness were excluded as the focus of the study is more on prevention of poor mental health and fostering good mental health than it is on cure or condition management.

#### Intervention

Studies had to encompass at least one NBI integrating explicit and purposeful nature contact, either encompassing blue or green space. This could be accomplished through direct nature exposure to an authentic natural setting (e.g., being in a park, being surrounded by indoor plants, having natural window views) or through indirect nature contact such as technological nature (e.g., acoustical and visual features). Studies that solely investigated existing restorative design features and qualities (e.g., plant density) within the working environment without manipulating these features were excluded. Exercise- and physical activity–based interventions that took place outdoors only met the inclusion criteria when natural features (e.g., trees) were present and sufficiently described (i.e. met green exercise definition).

Within this systematic review, *nature* is generally defined as spaces including elements of living systems with flora and fauna across a range of scales and degrees of human management, from a minor urban park through to relatively untouched wilderness (83). The term *green space* describes vegetation (e.g., trees, parks, forests, grass, etc.), whereas *blue space* prominently features visible surfaces of water (e.g., lakes, rivers, coastal water) (13, 84). Nature contact includes various dimensions and differs in spatial scale, frequency, proximity, the sensory pathway (e.g., visual vs. auditory experience), the person’s activities and awareness in a natural environment (73).

#### Comparison

Comparison conditions had to be no intervention-control conditions or to display a clear contrast to nature, encompassing equivalent interventions in a nonnatural environment (e.g., built or urban environment, indoors. with no visual access to nature elements such as a view on nature from a window).

#### Outcomes

To be eligible, studies were required to report quantitative data on mental health (e.g., optimal functioning) or well-being (e.g., experience of positive emotions) using questionnaires. Measuring psychophysiological indicators signaling stress responses (e.g., blood pressure) were desirable as secondary outcomes, but not mandatory as an inclusion criterion.

#### Study Design

Eligible study designs included: randomized controlled trials (RCTs), quasi-RCTs, controlled trials (CTs), randomized cross over trials (RXT), quasi-RXTs, and crossover trials (XTs). Observational studies and studies without a control or comparison group were not eligible. Only peer-reviewed fully published research was included. Papers that contained conference proceedings, dissertations or project description reports and book chapters were excluded. Additionally, secondary sources and study designs such as systematic reviews, meta-analyses and literature (narrative) reviews were also excluded. There were no imposed restrictions publication period or language of the publication.

### Search Strategy

Files (.ris format) containing the exported search results were saved and imported into the Rayyan web tool (85) for removal of duplicates and title and abstract screening. After deduplicating, titles and abstracts were independently screened by two authors (SG, JBB). The percentage of abstracts for which the two reviewers decided to exclude differed (88% and 91.8%), resulting in 26 articles of conflict. These discrepancies were resolved through discussion with a third researcher (TEM), resulting in two additional excluded articles. Following this first screening phase, full-text copies from articles that appeared to fulfill the inclusion criteria or where uncertainty still existed were retrieved. Two authors (SG, TEM) individually determined the final eligibility based on the full-text.

Subsequently, differences in eligibility assessment of two papers were adjudicated through consensus procedure or when necessary with the involvement of a third researcher (GC). The reference lists of included articles were scrutinized to identify further relevant studies. Throughout the whole process, the prespecified inclusion and exclusion criteria were applied.

### Assessment of Risk of Bias

The Revised Cochrane risk-of-bias tool for randomized trials (RoB 2) was employed to gauge the risk in the findings of included studies on the following clustered outcome categories: (*i)* mental health indices; (*ii)* cognitive ability; (*iii)* recovery and restoration; (*iv)* work and life satisfaction; and (*v)* psychophysiological indicator of health. It addresses five bias domains: randomization, deviations from intended interventions, missing outcome data, measurement, selection of reported results (86). Each domain was judged as *low*, *some concerns*, or *high risk* based on responses to signaling questions, resulting in an overall bias judgment for the specific study outcome being assessed. Two authors (DD, GC) independently determined the risk-of-bias, with any disagreements resolved by a third researcher (SG).

### Data Extraction

A data extraction table was developed (SG). The extracted data included the lead author, year of publication, country, theoretical framework, study design, methodology, population characteristics, intervention type and description (i.e. setting, nature component, daytime), as well as reported outcomes. One reviewer (SG) abstracted the aforementioned information, which were double-checked by a second reviewer (JBB).

Due to the paucity of research addressing the question of interest, coupled with the heterogeneous nature of the clinical, methodological, and statistical approaches employed, it was not feasible to pool data across studies to calculate a single effect estimate as initially planned. Thus, a narrative synthesis was conducted with the aid of guidance documents (87). The incongruous nature of the data across the studies led the authors to conclude that a quantitative meta-analysis would potentially be misleading and inappropriate (88). After preliminary analysis of the design and participant characteristics across studies, the studies were organized according to intervention type and outcomes incorporating a risk-of-bias assessment.

## Results

**Figure 1:**
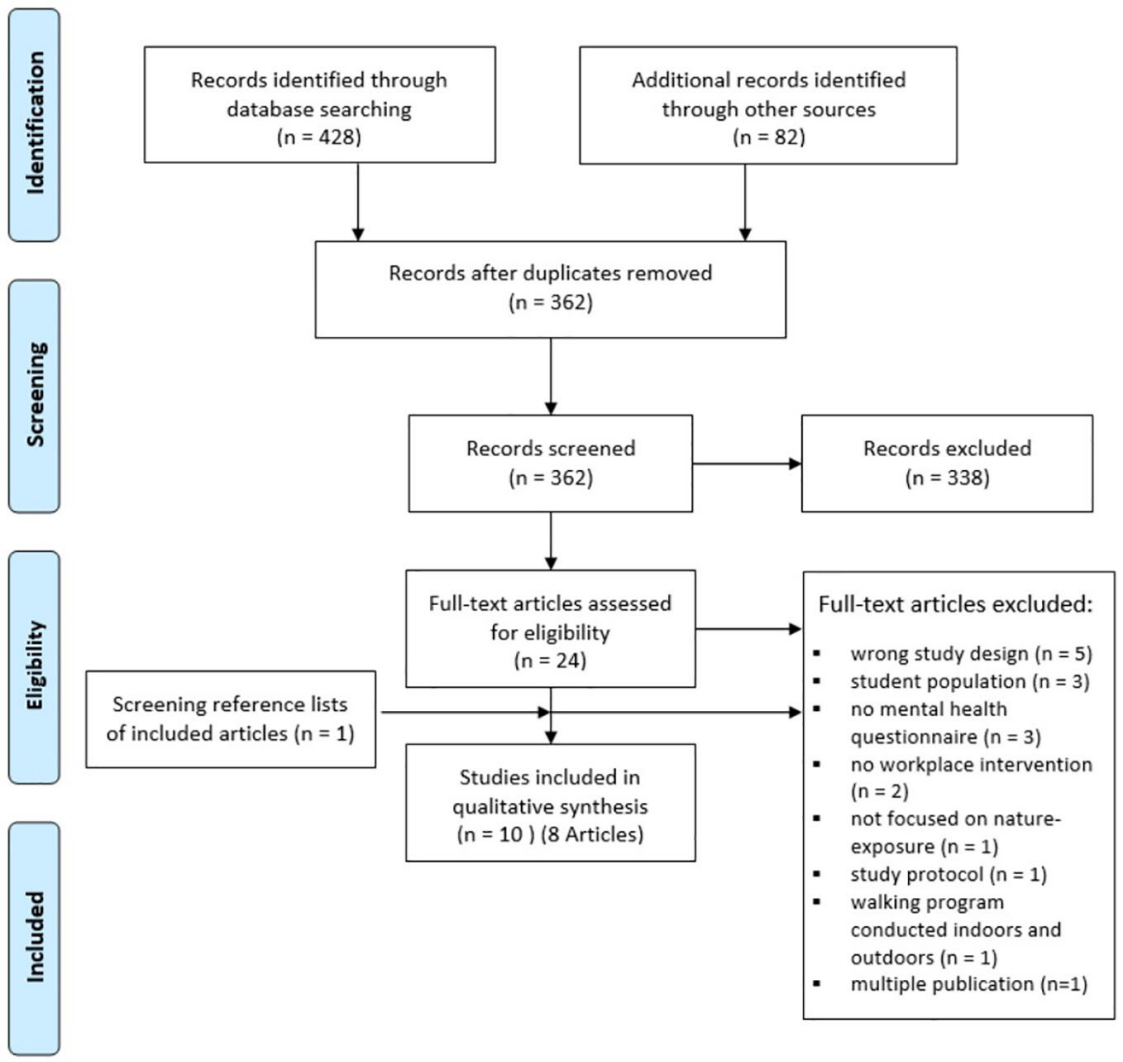
Preferred Reporting Items for Systematic Reviews and Meta-Analyses (PRISMA) flow diagram of study selection and identification.

A PRISMA flow diagram was developed to summarize the selection of the studies retrieved for the review process (see **Figure 1**). Briefly, of the 510 references obtained from the search, 24 articles remained after title and abstract screening. One article (89) was translated from Korean into English with the help of an international student with Korean as their first language, and one other article (90) was translated from Japanese with the help of a researcher with sufficient knowledge of the language. One abstract revealed to be solely published as a conference abstract; the authors were contacted in order to ask whether the data had been published or in press as a full publication, but a full-text copy could not be obtained. Five other articles did not contain sufficient information (e.g., characteristics of the environment with respect to the presence of natural elements), thus the authors were contacted for complementary information. Scrutiny of the reference lists of included publications resulted in one additional hit (91). Eventually, nine articles, containing 10 NBIs involving 611 employees overall, met the inclusion criteria. One article (92) comprised three independently conducted NBIs (further referred to 92). Two NBI trials had multiple publications (91, 93–96). For the first NBI trial with two publications, Calogiuri et al. (93) is the primary reference, as this article comprised quantitative analysis of well-being indicators (e.g., affect). However, additional outcomes regarding psychophysiological indicators in Calogiuri et al. (91) are reported. De Bloom et al. (94) and Sianoja et al. (95) refer to the same NBI, which was conducted as two independent RCTs phases with different participants (in spring and fall), but reported a different set of outcomes (94, 95). The third publication was excluded due to the absence of a questionnaire regarding mental health and well-being (96). The article by de Bloom et al. (94) analyzed and interpreted the results of both RCTs phases separately, while Sianoja et al. (95) pooled the RCTs together. Thus, due to an earlier publication date and the importance of weather and season variabilities in NBIs, it was decided to include the study by de Bloom et al. (94) as a primary reference and to report the RCT phases as two independent RCTs (further referred to as 94). However, two outcomes of the study by Sianoja et al. (95) are reported in this systematic review: the theoretical framework and the additional analyzed outcomes perceived stress and concentration. Here, it is necessary to bear in mind, that Sianoja et al. (95) included participants of both studies in de Bloom et al. (94). The remaining articles described one NBI each (89, 90, 93, 97, 98). For an overview of study and participant characteristics, see **Table 2**.

**Table 2:** Study and participant characteristics.

Study (Author, year)	Country	Population	Overall N (% f)	Type of NBI	Dur. in (min)	Freq. per week	Length in weeks	Program	Type of nature	Control condition	Comparison/experiment 2 condition
Bang et al. (89)	South Korea	office workers (faculty members + researchers in Seoul)	60 (92.6)	green exercise	40	2	5	urban forest-walking program	“palace area,” park	no instruction	–
Brown et al. (97)	United Kingdom	office workers (desked based jobs in financial sector, one company at two sites)	94 (21.3)	green exercise	20	2	8	nature walking, circular walking route (approx. 2km), individually or with others	trees, spaces of maintained grass, public footpaths, country lanes	waiting control group	built walking group (BW): paved footpaths adjacent to roads, housing estates, industrial area
Calogiuri et al. (91, 93)	Norway	office workers (mainly office-based work, municipality employees, sedentary or moderately active)	14 (50.0)	green exercise	45	1	2	nature exercise program consisting of 2 parts: bicycling + strength session	forest area, grass-yard	–	bicycling + strength session in gym-hall, no visual nature contact, artificial lights, natural light filtered
de Bloom et al. (94)	Finland	diverse (knowledge- intensive + emotionally demanding jobs: public sector, administration, media, health care, finance, engineering)	83 (89.2)	green exercise coupled with nature savoring	15	5	2	park walk in nearest park, alone or in a group, instructed to pay attention to surroundings (savoring)	park	usual break activities	relaxation techniques: release-only version of progressive muscle relaxation, deep breathing + acceptance
de Bloom et al. (94)	Finland	diverse (knowledge- intensive + emotionally demanding jobs public sector, education, engineering)	70 (90.0)	green exercise coupled with nature savoring	15	5	2	park walk in nearest park, alone or in a group, instructed to pay attention to surroundings (savoring)	park	usual break activities	relaxation techniques: release-only version of progressive muscle relaxation, deep breathing + acceptance
Largo-Wight et al. (98)	USA (Florida)	office workers (university staff)	37 (91.8)	nature savoring	10-15	5	4	daily sitting outdoor work break while focusing on natural elements (e.g., clouds, sky, sounds, trees, grass, water)	any place outdoors	daily indoor standard self-selected work break, but not work-related	–
Matsunag et al. (90)	Japan	medical personnel (doctors, nurses, care workers of elderly health care facility)	72 (77.8)	nature savoring	5	1	–	enjoying view for 5 min, while “sitting still”	roof top forest (outskirts), bird sound, lawn, trees, plants, herbs, background: mountains	–	1st floor asphalted outdoor parking lot, during experiment cars were banned
Nieuwenhuis et al. (92)	United Kingdom	office workers (international consultants)	67 (41.8)	green office space	–	–	3	enrichment of office space by indoor green spaces in open plan spaces, at least 2 plants in direct view	large-leafed plants (90cm)	no changes: lean minimalist office space: no plants in direct sight, on the same floor	–
Nieuwenhuis et al. (92)	Nether-lands	office workers (call center agents of a health insurance company)	81 (81.5)	green office space	–	–	2	enrichment of office space by indoor green spaces in open plan spaces, at least 1 plant in direct view	large-leafed plants (90cm)	no changes: lean minimalist office space: no plants in direct sight, on different floors	–
Nieuwenhuis et al. (92)	United Kingdom	office workers (international consultants)	33 (51.5)	green office space	–	–	–	while working on cognitive tasks: office room containing eight large plants, at least 3 plants in direct view	large-leafed plants (90cm)	no further additions to office space, lean office space	–

f, females; Dur, duration; Freq, frequency.

### Study Characteristics

Eight NBIs had a longitudinal (i.e. comprising several weeks) RCT design, of which three also employed a *three*-*arm* parallel-group design (94, 97). Six NBIs used a RCT design with randomization at the individual level, while two randomized at the group level (green office space design studies) (92). The interventions’ length ranged from 2 to 8 weeks. Although one pilot study implemented a brief intervention of merely two green exercise activities preceded by a exercise promotion workshop the previous week over a fortnight, it was classified as a longitudinal study due to the follow-up assessment of 2 and 10 weeks after the intervention (93). One other NBI applied a follow up measurement (after three and a half months) (92). Finally, one article included a preliminary cross-sectional survey on perceived feasibility of the NBI and a RXT investigating the effects of the actual NBI (90), and one employed an acute RCT design (92). The median sample size of studies was 70 participants and ranged from 14 (93) to 94 (97).

### Theoretical Frameworks

Seven publications were based on an environmental psychology perspective (excluding 90) with five explicitly referring to ART (92–95, 98) of which one further employed the *SRT* (93). Additionally, four papers utilized biological and evolutionary explanations for the beneficial effects of nature (90, 92, 95, 98) with one mentioning the biophilia hypothesis (98). Five papers drew upon *green exercise* research (89, 93–95, 97) with Bang et al. (89) not incorporating the term *green exercise* in their study. Moreover, five papers integrated an occupational health psychology perspective by referring to worksite health promotion efforts (93, 98) and enrichment of office spaces (92). A stronger theory driven approach was taken by de Bloom et al. (94) and Sianoja et al. (95) by encompassing the *Effort-Recovery Model*, *Conservation of Resources Theory*, and *Recovery Experiences*. Only one paper referred to the *Broaden-and-Built Theory* (95). Therefore, this publication is the only one that undertook the synthesis of environmental, work and positive psychology. Finally, only one article used the *information-motivation-behavioral skills model* (90).

### Country of Origin

The 10 NBIs took place in seven different countries. Three were conducted in the United Kingdom (92, 97) and two in Finland (94). The other NBIs were conducted in South Korea (89), Norway (93), United States (98), Japan (90) and the Netherlands (92). These countries differ largely on cultural dimensions like the value given to individualism, long-term orientation, masculinity and uncertainty avoidance (99–101). Due to this diversity, the countries cannot be considered as homogeneous in terms of society.

### Participant Characteristics

#### Demographics

In total, 611 employees participated across the ten studies. The mean age of the participants ranged from 28 to 49 years with an overall mean age of 45.5 (SD = 4.95) years. Most participants were female (mean % female = 67%; median = 78%). One exception was the pilot study by Calogiuri et al. (93) with an exact gender split of 50%. However, the observed gender imbalance may be due to gender segregation in the labor markets, for example, financial sector (97), and education (89). Studies lacked an explicit reporting of ethnicity, with only two studies (97, 98) providing ethnicity data. In these two studies there was a predominance of Caucasians (mean = 82%). Only one publication including two experiments (94) reported additional sociodemographic factors such as educational level and household, revealing an overrepresentation of participants cohabitating with children and possessing a master’s degree or higher.

#### Occupations

Seven studies comprised office workers, specifically: university staff (89, 98), finance employees (97), municipality employees (93), call center agents (92), and consultants (92). The study by Matsunaga et al. (90) involved doctors, nurses and care workers at an elderly health care facility (90). The remaining two studies (94) encompassed diverse employees in knowledge-intensive and emotionally demanding jobs from different companies and work sectors (public sector, administration, media, health care, finance, and engineering). Again, these studies were the only studies that captured work-related factors (e.g., permanent work contract, supervisory position, weekly work hours, work type such as blue- or white-collar worker, and tenure). Only Nieuwenhuis et al. (92) collected the number of work years for the company in study (a) and (b), too (92).

#### Intervention Types

Among all studies, different NBIs were identified and grouped into three categories: (*i)* green exercise, (*ii)* nature savoring, and (*iii)* green office space. Green exercise defines the synergy of physical activity and natural environment (65), whereas nature savoring is defined as mindfully noticing and attending nature while regulating the emotional impact of positive events by one’s cognitive or behavioral response (102, 103). The third category, green office space, comprises interior landscaping interventions that aim to transform the design of workplaces by enriching plants and other natural features.

##### Green Exercise Program and Type of Nature

Five green exercise interventions were conducted and varied considerably in their implementation (89, 93, 94, 97). One common attribute across four studies was the time of intervention—employees’ lunch break. Only the participants in the study by Calogiuri et al. (93) completed their green exercise in the afternoon following a regular working day. Bang et al. (89) implemented an urban forest-walking program under the direction of the researcher, which took place in a “palace area” with park. Brown et al. (97) provided a more detailed vegetation description including trees, spaces of maintained grass, public footpaths and country lanes. The employees could choose whether to walk the circular route (approximately 2 km) alone or in a group. Similarly, in de Bloom et al. (94) and Sianoja et al. (95) participants could decide whether to walk independently or collectively. The researchers introduced a green exercise intervention slightly coupled with a nature savoring component; the park walk instructions prompted participants’ to pay attention to their surroundings and to avoid talking. The studies lacked a sufficient description of the type of nature (i.e. “nearest park”). Calogiuri et al. (93) was the only trial that implemented a green exercise program consisting of bicycling and strength training with an experienced instructor as opposed to walking programs. The cycling part was performed in a forest area, whereas the subsequent strength session was held in a grass yard. This publication was the only one that provided photographs of the natural settings.

*Exercise Intervention Characteristics—*The intervention length, and session duration, frequency, and intensity varied widely. For example, the program of both de Bloom et al. (94) experiments consisted of a 15 min slow, low-intensity walk on every working day (i.e. 5 days a week) within a 2-week intervention period (i.e. 10 sessions overall). Other walking programs employed a 5-week intervention with a duration of 40 min biweekly (i.e. ten sessions overall) (89) and an 8-week intervention with a duration of 20 min biweekly (i.e. 16 sessions overall) (97). Neither study reported the physical activity intensity. The average duration of green exercises ranged from 15 to 45 min (mean = 27 min). Calogiuri et al. (93) had participants exercise for 45 min (i.e. cycling for 25 min followed by a 20-min strength session using elastic rubber bands with handles). This exercise was performed at a moderate-intensity on 2 days over 2 weeks. To assess the reporting quality of eligible exercise interventions we used the 16-item Consensus on Exercise Reporting Template [CERT; (104)]. All five exercise interventions lacked sufficient information, resulting in a total score of nine (max. score = 19), respectively ten (93), after applying the CERT (JBB). Domains that were not addressed included the detailed description of motivation strategies, adverse events, and the extent to which exercise was tailored.

*Environmental Conditions—*Overall, weather conditions were poorly assessed and described. All five studies reported the intervention months: October–November (89), May–July (97), September (93, 94), and May (94). Three trials documented additional environmental conditions: 8°C –10°C with sunny conditions on the first day, overcast on the second day of green exercise (93), an average temperature of 15°C in spring, no precipitation and mostly sunshine, with daily temperatures up to 28°C (94) and an average of 14°C in fall with again no precipitation and mostly sunshine (94).

*Comparators—*There were substantial divergences in comparison conditions. In one study, the control group was given no instructions and told to have a regular daily life (89). Calogiuri et al. (93) compared green exercise with exercising indoors (i.e. gym hall) under identical conditions regarding duration, frequency, and intensity. Visual contact with nature was avoided and natural light was filtered. In two RCTs the control group was instructed to maintain their usual break activities. The other comparison, a second experimental condition, consisted of relaxation techniques, namely a release-only version of progressive muscle relaxation, deep breathing, and acceptance of the here-and-now (94). The remaining trial also employed two comparison conditions: a waiting control group and a built walking group without access to nature, comprised paved footpaths adjacent to roads (97).

*Feasibility and Adherence—*The validity of RCTs evaluating exercise programs depends strongly on participants’ adherence rates, which reflects the attendance and compliance to the prescribed sessions. This varied largely across NBIs. For instance, in Calogiuri et al. (93), which consisted in an exercise promotion workshop followed by two green exercise sessions over a fortnight, all participants completed the NBI. The adherence to the study protocol in the two NBIs described in de Bloom et al. (94) was still fairly high, with 76% engaging in the green exercise condition or relaxation technique (comparison group) at least eight out of ten times within a 2-week time frame, respectively 72%. On the other hand, the longer intervention described in Brown et al. (97) reported quite a lower adherence rate of merely 43% in the nature and 42% in the built walking condition over an 8-week intervention.

##### Nature Savoring

Two studies implemented a nature savoring intervention employing markedly different designs.

*Savoring Intervention Characteristics—*In a 4-week longitudinal trial, office workers took a self-selected daily outdoor break during the work day for 10–15 min (a total of 20 breaks) while aiming attention at natural elements such as clouds, sky, trees, bird sounds, grass, vegetation, water, or fountains (98). The environment was no further specified than “any place outdoors.” In the within-subjects design study by Matsunaga et al. (90), medical staffs were exposed to a fourth story rooftop forest view on a single occasion (five min) while “sitting still in a wheelchair.” This rooftop was covered with lawn, trees, and plants with mountains in the background.

*Environmental Conditions—*In Matsunaga et al. (90), the environmental conditions were reported to be sunny with an average temperature of 22.8°C and an air humidity of 37.4%. Weather conditions were not reported in Largo-Wight et al. (98).

*Comparators—*In Largo-Wight et al. (98), the control group undertook a daily indoor standard work break, which was self-selected in terms of time and location, but should not be work-related. In Matsunaga et al. (90), the comparison environment consisted in observing a first-floor outdoor parking lot. Order effects were minimized by creating sex- and age-matched groups starting either with the nature or comparison condition. Pictures of both environments were provided in the publication.

*Feasibility and Adherence—*Matsunaga et al. (90) reported no dropouts, although it should be noted the NBI (and its comparison) took place during one single day. In Largo-Wight et al. (98), prior to conducting the actual NBI, an online survey investigating the perceived feasibility of the proposed intervention was distributed among office staff. Responses revealed that participants perceived the study protocol to be feasible (74%), practical (80%), and worthwhile (83%). This was later confirmed when, actual the actual NBI, all employees reported a high compliance (88% not missing any assigned work break).

##### Green Office Space

All three NBIs in Nieuwenhuis et al. (92) demonstrated the enrichment of open plan office spaces by incorporating indoor green spaces using large-leafed plants (90 cm tall). The plants were continuously present over either 3 weeks (92) or 2 weeks (92). However, data were collected at baseline and after 8 (92) and 5 weeks (92), and the plants were installed for each intervention length followed by subsequent postintervention assessment. Each employee had at least one (92) or two plants (92) in direct view. The control group worked either on the same floor (92) or on a different floor (92) and both experienced no working environment change and continued performing their job in a lean, minimalist office space. In the third study, consultants worked on cognitive tasks at the end of the working day in a randomly assigned experimental condition green (at least three plants in direct view) vs. lean office space (92).

### Risk of Bias

**Figure 2:**
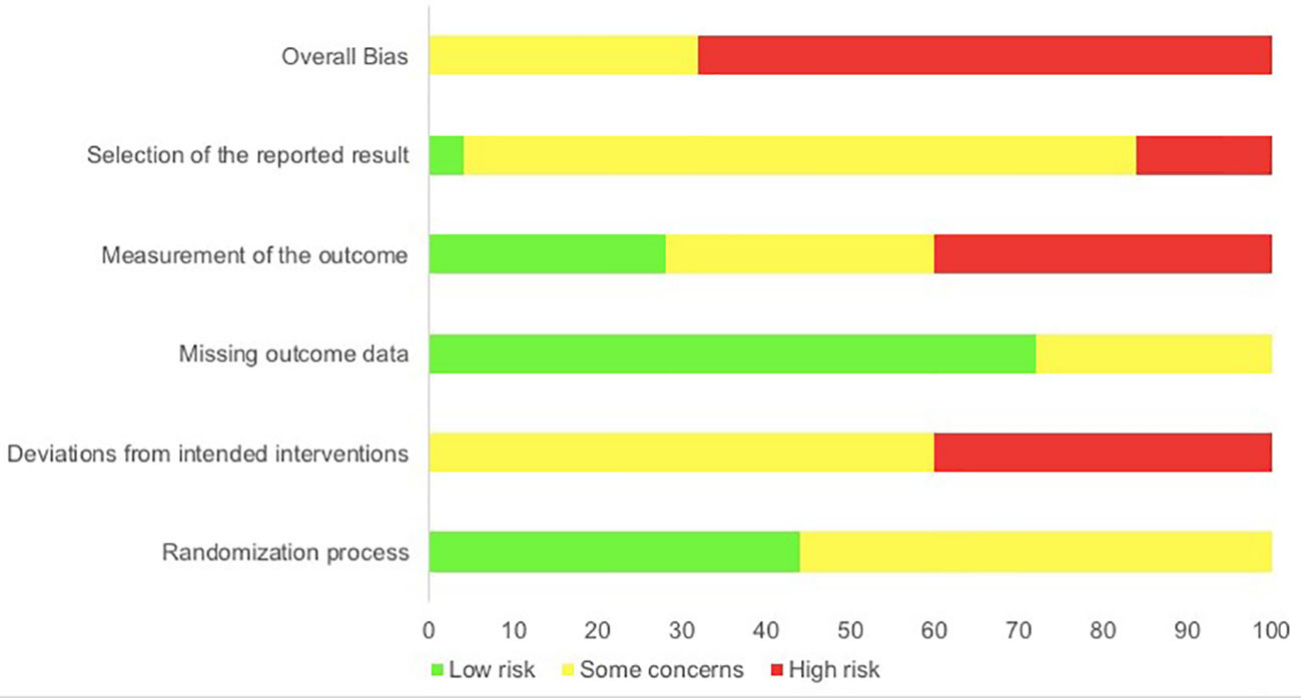
Percentages summary of risk-of-bias assessment using the RoB 2 tool.

**Figure 3:**
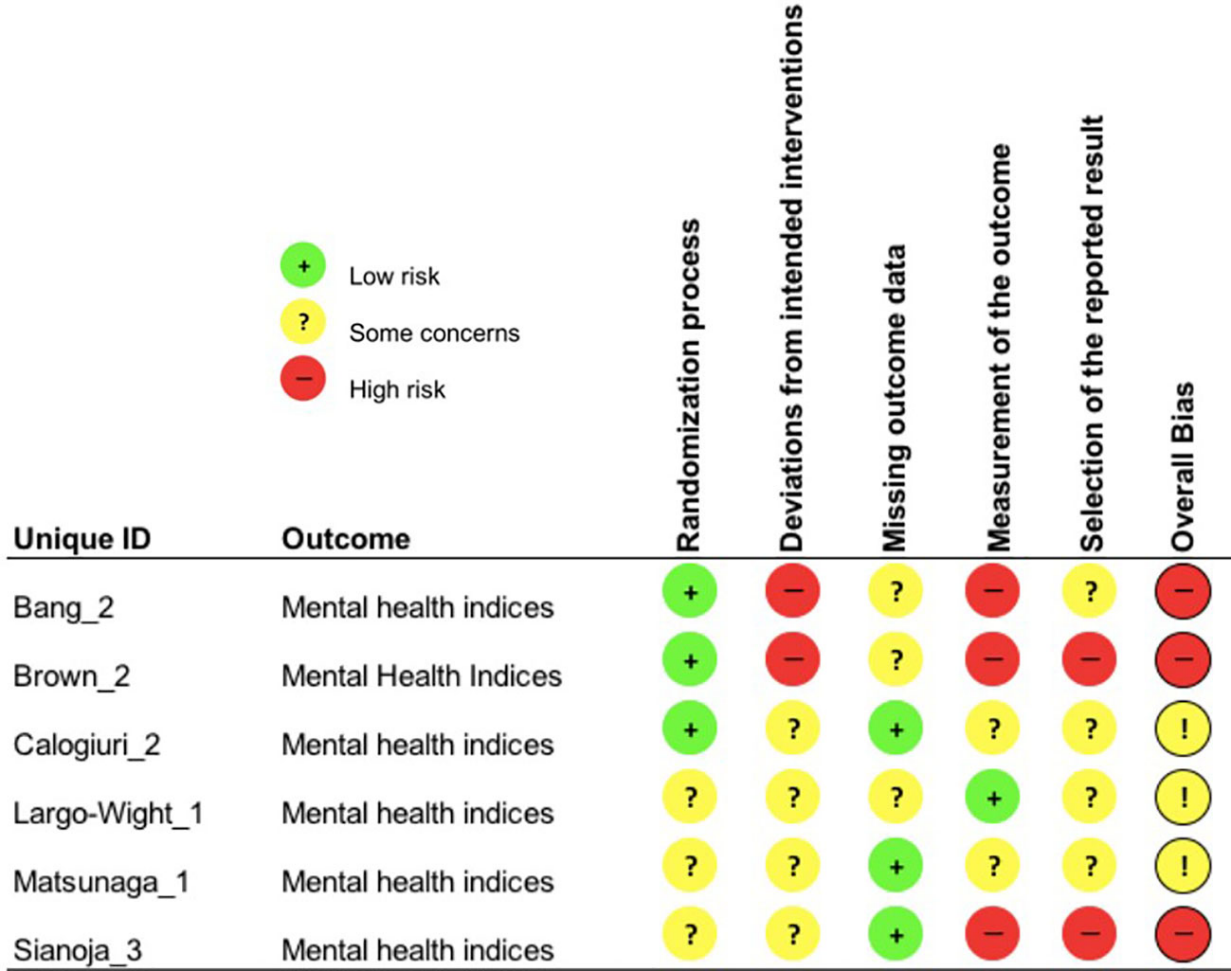
Risk of bias assessment for the category mental health indices.

**Figure 4:**
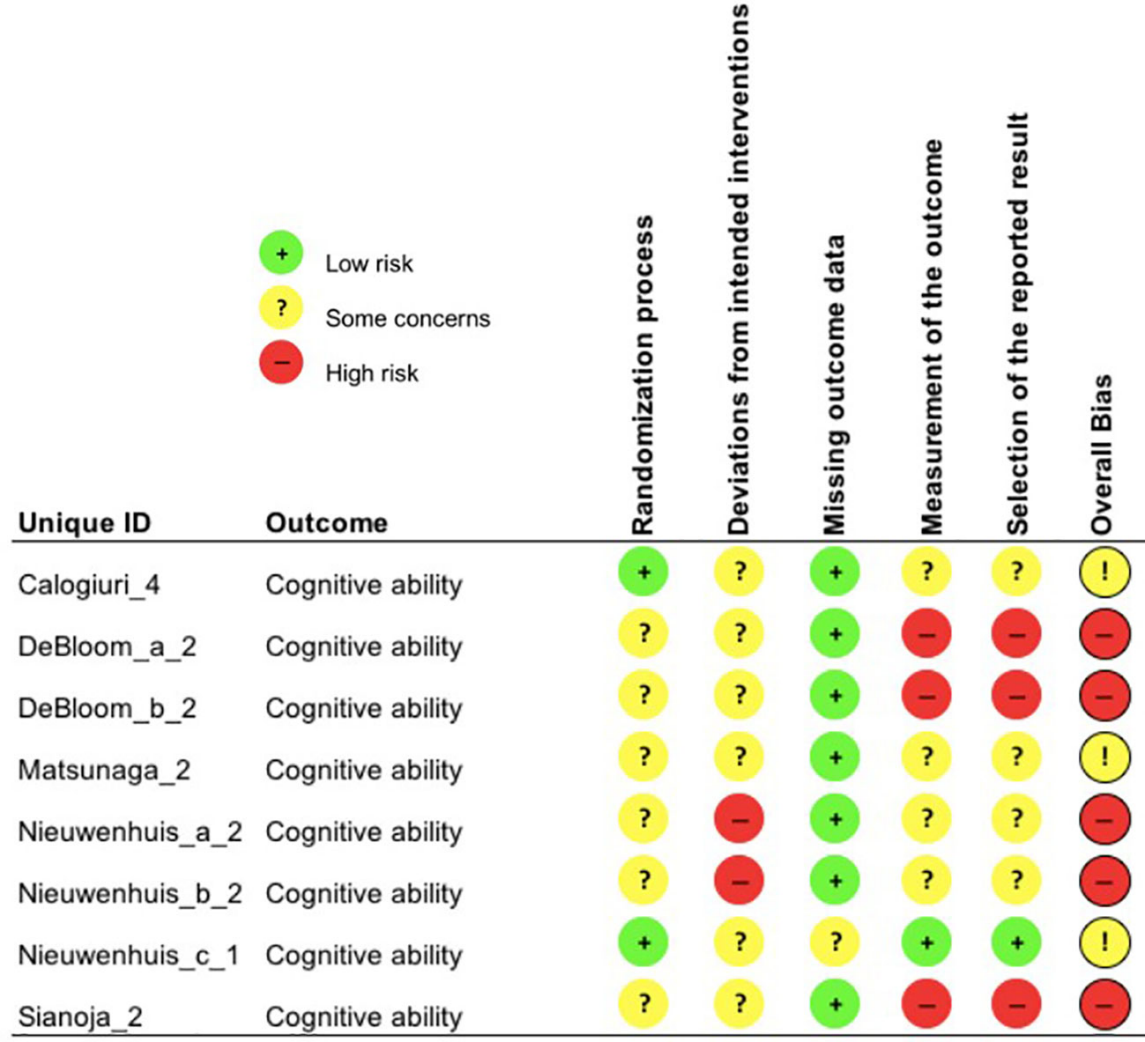
Risk of bias assessment for the category cognitive ability.

**Figure 5:**
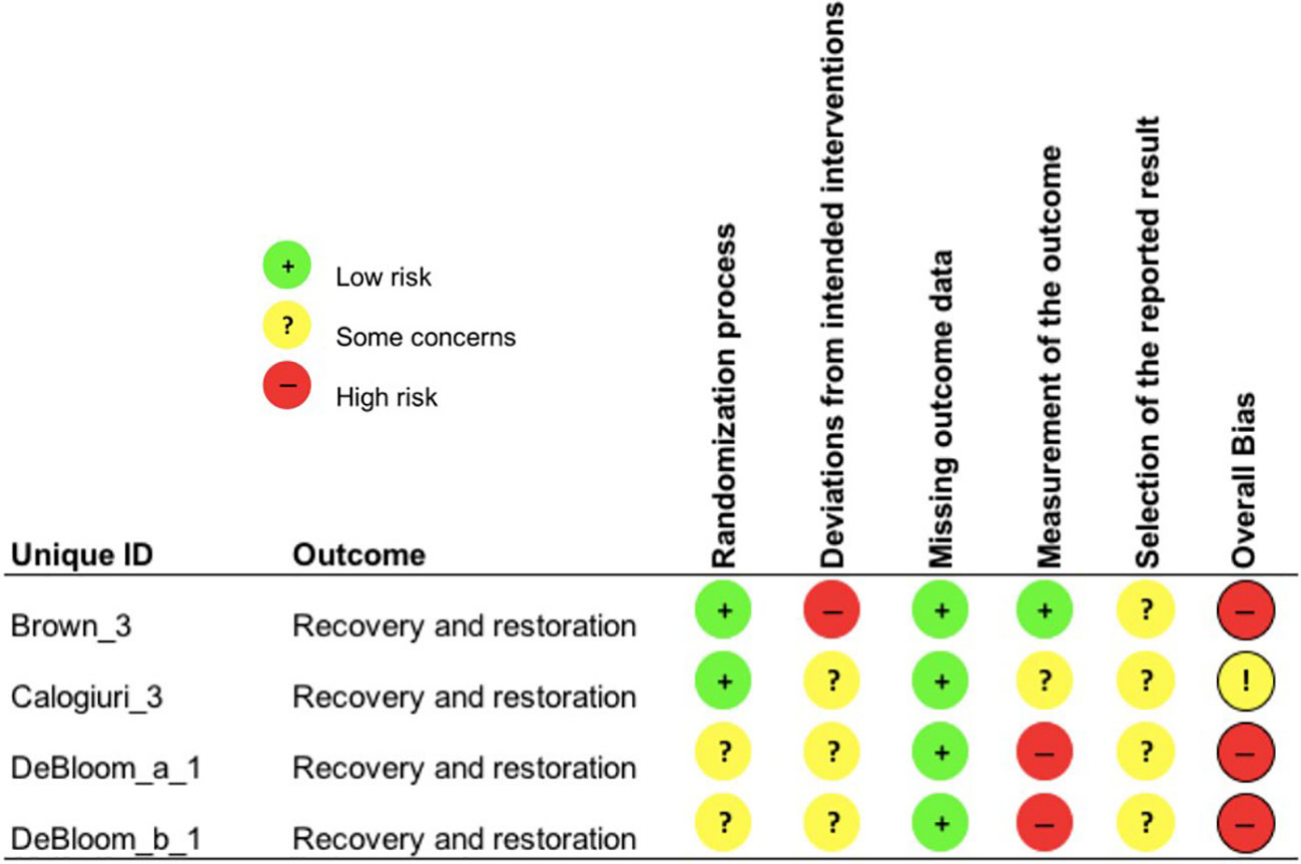
Risk of bias assessment for the category recovery and restoration.

**Figure 6:**
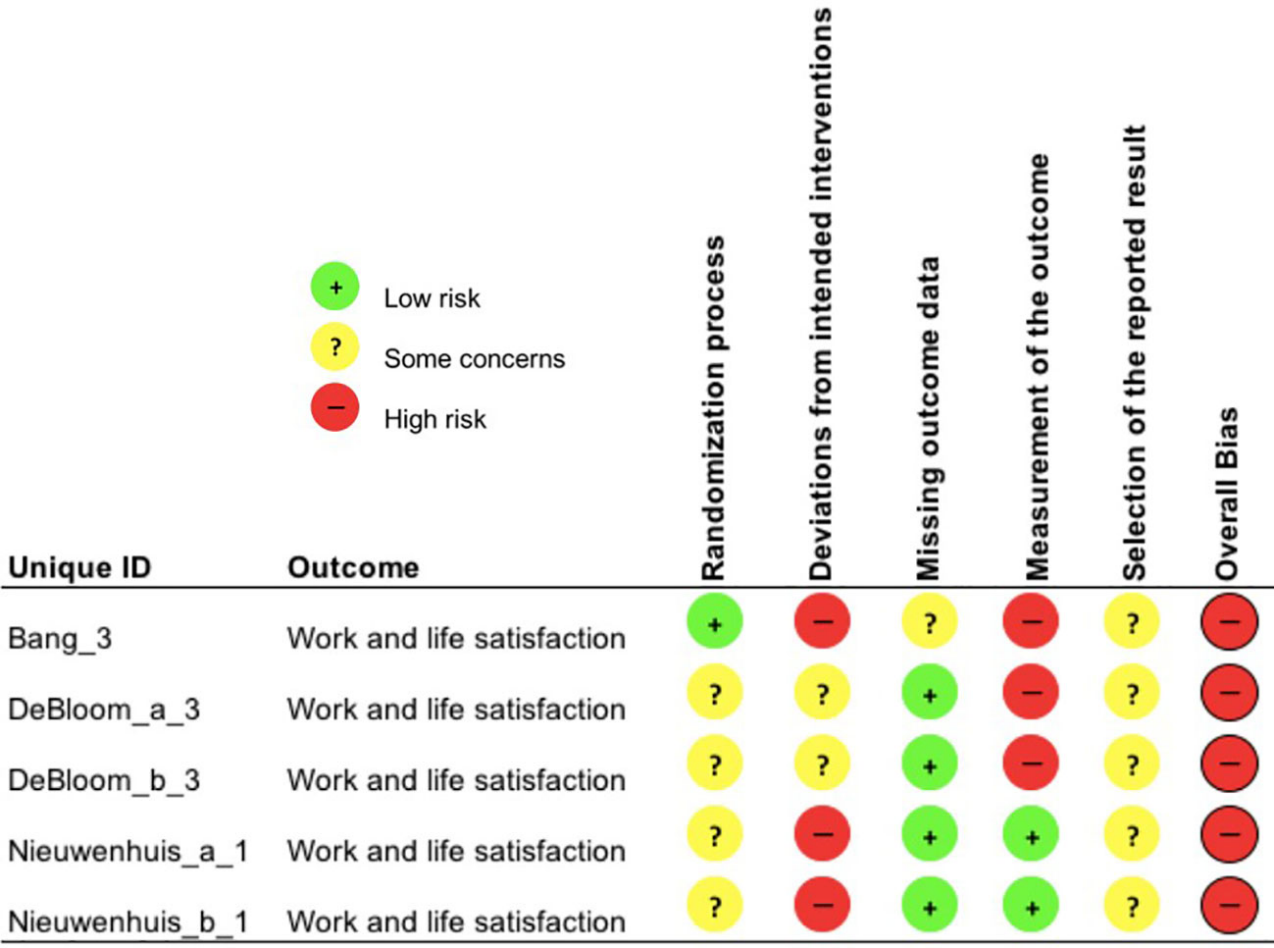
Risk of bias assessment for the category work and life satisfaction.

**Figure 7:**
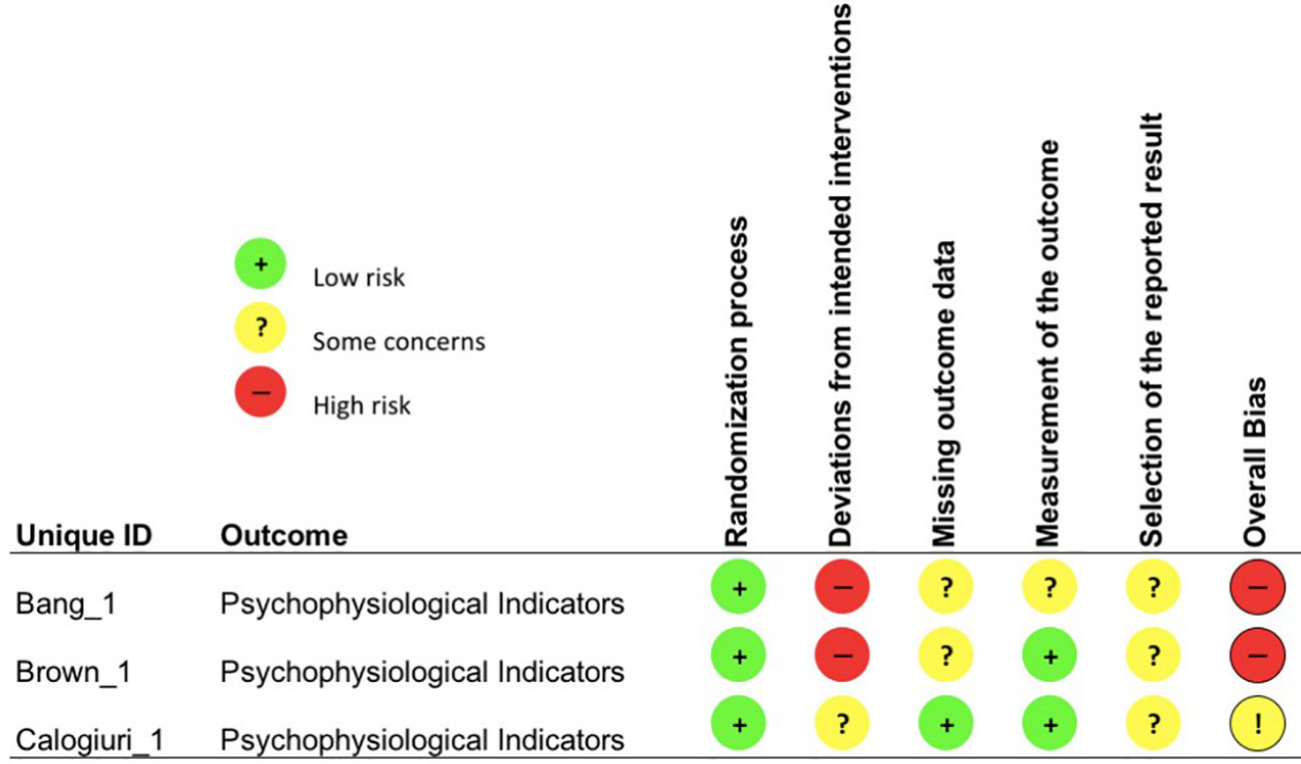
Risk of bias assessment for the category psychophysiological indicators of health.

Each study outcome was categorized into one of the five clustered outcome categories [(*i)* mental health indices; (*ii)* cognitive ability; (*iii)* recovery and restoration; (*iv)* work and life satisfaction; and (*v)* psychophysiological indicators of health], assigned a unique ID and assessed for risk-of-bias (e.g., Bang_1; see **Table 3**). Thus, multiple RoB 2 assessments were conducted for each publication, in line with such clustering. An overall overview of the outcomes of the RoB 2 assessment is presented in **Figure 2**, whereas, the outcomes of the RoB 2 for the different outcome categories can be found in **Figures 3**–**7**, with “+” indicating *low risk*, “?” or “!” *some concerns* and “-” *high risk*.

**Table 3:** Measured outcomes for each study with unique ID categorized to clustered outcome categories.

Clustered outcome categories					
Study (Author, year), type of NBI	Psychophysiological indicators of health	Mental health indices	Work and life satisfaction	Recovery and restoration	Cognitive ability
Bang et al. (89), GE	**Bang_1** Subjective PA, BMI, BC, BP, BD	**Bang_2** Depression (BDI)	**Bang_3** Quality of life (GHQ/QL-12)		
Brown et al. (97), GE	**Brown_1** Objective PA, BMI, HR, HRV, BP, CVD risk, Aerobic fitness, PH (SF-8)	**Brown_2** General mental health state (SF-8)		**Brown_3** Stress response + recovery (HR, HRV)	
Calogiuri et al. (91, 93), GE	**Calogiuri_1** CAR, BP serum cortisol, PA	**Calogiuri_2** Mood/affect: positive + negative Affect + tranquility (PAAS)		**Calogiuri_3** Perceived restorative-ness: fascination + being away (PRS)	**Calogiuri_4** Fatigue (PAAS)
de Bloom et al. (94), GE			**DeBloom_a_3** Job satisfaction (1 item)	**DeBloom_a_1** Restoration (1item) and recovery: RX + PD + enjoyment (3 items in total, 2 from REQ)	**DeBloom_a_2** Fatigue (1 item)
de Bloom et al. (94), GE			**DeBloom_b_3** Job satisfaction (1 item)	**De Bloom_b_1** Restoration (1 item) and recovery: RX + PD + enjoyment (3 items in total, 2 from REQ)	**DeBloom_b_2** Fatigue (1 item)
Sianoja et al. (95), GE		**Sianoja_3** Perceived stress/strain (1 item)		**Sianoja_1** Recovery	**Sianoja_2** Fatigue + concentration (1 item)
				RoB not performed as these measurements are the same assessed for de Bloom 2017	
Largo-Wight et al. (98), NS		**Largo-Wight_1** Perceived stress (PSQ)			
Matsunaga *et al. (90), NS		**Matsunaga_1** State anxiety (STAI), mood + subconstructs: tension-anxiety, depression-dejection, anger-hostility, vigor, confusion (POMS)			**Matsunaga_2** Fatigue (POMS)
Nieuwenhuis et al. (92), GO			**Nieuwenhuis_a_1** Workplace satisfaction (4 items)		**Nieuwenhuis_a_2** Concentration (1 item), Subjective productivity (2 items)
Nieuwenhuis et al. (92), GO			**Nieuwenhuis_b_1** Workplace satisfaction (4 items)		**Nieuwenhuis_b_2** Concentration (1 item), disengagement (6 items), objective productivity
Nieuwenhuis et al. (92), GO					**Nieuwenhuis_c_1** Concentration, cognitive performance (processing + vigilance tasks)

*For this RXT trial additional required considerations for the RoB assessment were followed. GE, Green Exercise; NS, Nature Savoring; GO, Green Office; PA, Physical Activity; BMI, Body Mass Index; BC, Body Composition; BP, Blood Pressure; BD, Bone Density; BDI, Beck Depression Inventory; GHQ/QL, Quality of Life Scale of the General Health Questionnaire; HR, Heart Rate; HRV, Heart Rate Variability; CVD, Cardiovascular Disease; PH, Physical Health; SF-8, Short Form Health Survey; CAR, Cortisol Awakening Response; PAAS, Physical Activity Affective Scale; PRS, Perceived Restorativeness Scale; RX, Relaxation; PD, Psychological Detachment; REQ, Recovery Experience Questionnaire; PSQ, Perceived Stress Questionnaire; STAI, State and Trait Anxiety Inventory; POMS, Profile of Mood States.

Overall, not one study outcome out of 26 outcome measurements assessed displayed a low overall risk-of-bias. *Some concerns* (32% of outcomes) and *high risk-of-bias* (68%) were present in all outcomes due mainly to high-risk assessments in selection of the reported result (16%), measurement of the outcome (40%) and deviations from intended interventions (40%). Just two bias domains displayed no *high risk-of-bias:* missing outcome data (*low*: 72%, *some concerns*: 28%) and randomization process (*low*: 44%, *some concerns*: 56%). A summary of the risk-of-bias assessments for each clustered outcome category is presented below, alongside the respective findings for the respective category.

#### Mental Health Indices

##### Risk of Bias

Three study outcomes displayed a high overall risk of bias, due to contamination between intervention and control, respectively comparison group (i.e. working in the same building and discussing interventions with each other) (*deviations from intended intervention*; Bang_2, Brown_2) and very poor adherence and the failure to implement the intervention as planned (i.e. less than 50% of participants fully complied with the intervention) (Brown_2). Other reasons were high knowledge of the assigned intervention and its likelihood to influence employee-reported outcomes (*measurement of the outcome*; Bang_2, Brown_2, Sianoja_3) and trial protocol submission after data collection was finished (*selection of the reported result*; Brown_2). According to a trial protocol multiple indices of mental health measurements (e.g., perceived stress scale) and time points should have been collected (105), but were not reported or analyzed, without justification. Moreover, the statistical significant effect found for this outcome, alongside with a pooled sample size of two RCTs, suggest high a risk of selective *reporting of the results* (Sianoja_3). The other three mental health indices outcomes were judged to raise *some concerns* in at least three (Calogiuri_2) or four RoB 2 domains (Largo-Wight_1, Matsunaga_1).

##### Study Outcomes

Bang et al. (89) found no statistically significant difference in depression between green exercise and control group (*t* =.93, *p* =.358). Whereas in the nature savoring condition in Matsunaga et al. (90) female participants displayed significant decreases on the subscales depression-dejection (*p* < .01) and tension-anxiety (*p* < .01). Moreover, the mean values of state anxiety showed a significant reduction *(p* < .01) to 34.6 ± 8.1 (43.4 ± 8.4 for control) for male employees and to 36.3 ± 10.2 (45.8 ± 8.8) for female employees. Particularly, in women with a low- to medium trait anxiety, the state anxiety significantly (p < .01) decreased to a “very low” anxiety state after nature savoring, and in high trait anxiety females to a “low” anxiety state (p < .01). Across both genders, the scores revealed a significant increase in vigor *(p < .01)* postintervention. The subscales anger-hostility and confusion demonstrated no significance. In the study by Brown et al. (97), the green exercise participants displayed an increased self-reported mental health mean score by 2.7 above baseline score (95% *CI* 0.0–5.4) while the control group (−3.3; 95% *CI* –6.3–0.3) and built-walking group (−0.3; 95% *CI* −4.3–3.8) did not. Municipality workers in the green exercise condition from Calogiuri et al. (93) demonstrated only a marginally significant higher positive affect (*p* =.06) postexercise. Yet, these employees reported greater engagement with nature and scored higher on positive affect (*p* =.02) than the indoor group over a 10-week follow-up period. No significant difference was found for tranquility. Negative affect was excluded from the dependent variables due to poor normality. Office workers in the nature savoring condition displayed a significant lower posttest stress score (*p* =.041) compared to the control group, in a main effects ANCOVA model controlling for baseline stress (98). Interactions and additional covariates (e.g., sex) were not significant. Sianoja et al. (95) collected day-level data twice a week for 5 working weeks (NBIs in week 2 and 3). Green exercise predicted lower levels of afternoon strain on the within-person level (*β* = −.34, *SE* =.17, *p* < .05). However, the beta coefficient in the comparison group (relaxation exercises) revealed to be even greater (*β* = −.60, *SE* =.18, *p* < .01). Thus, employees reported lower levels of strain before leaving work, when they had engaged in green exercise or relaxation techniques during lunchtime. After the inclusion of detachment (i.e. recovery experience) and enjoyment as mediator variables, the main effect for green exercise remained only marginally significant.

#### Cognitive Ability

##### Risk of Bias

The majority of cognitive ability outcomes (n = 5) showed a high overall risk of bias due to very large number of dropouts (Nieuwenhuis_a_2, Nieuwenhuis_b_2) and contamination between intervention and control group (*deviations from intended interventions*; Nieuwenhuis_a_2). Additionally, the rating of the domain *measurement of the outcome* resulted in a high overall risk of bias for three study outcomes: One-item measurements were used and elicit concern about reliability and validity, as well as the fact that employees had knowledge about assigned interventions, which was highly likely to influence their self-reported outcomes (DeBloom_a_2, DeBloom_b_2). The other three study outcomes on cognitive ability displayed *some concerns* overall (Calogiuri_4, Matsunaga_2, Nieuwenhuis_c_1).

##### Study Outcomes

Green exercise participants showed a trivial decrease in afternoon fatigue during the intervention period (*d* = −19, *p* < .05) and postintervention (*d* = −.22) compared to baseline (94). Whereas the control-group (i.e. no intervention) showed an increase in afternoon fatigue during the intervention period (*d* =.27). Surprisingly, green exercise participants reported an increase in fatigue in the evening (*d* = −.22). Repeated measures ANOVAs revealed no significant effects for group × time interaction on the between-subjects level. Participants in the second study (94) displayed lower fatigue directly after the lunch break (*d* =.52), in the afternoon during (*d* =.54, *p* < .05) and at the end of the intervention period (*d* =.29, *p* < .05). Afternoon fatigue in the control group increased after the intervention period (*d* = −.30). No effects were found for evening fatigue. Matsunaga et al. (90) reported that after viewing the rooftop forest (nature savoring), employees’ fatigue significantly decreased (*p* < .01).

Employees in the green office condition in Nieuwenhuis et al. (92) self-rated their ability to concentrate higher after the introduction of plants (*F*_1,65_ = 11.11, *p* =.001). In contrast, no significant difference over time was found in the lean condition (*F*_1,65_ =.63, *p* =.431). Thus, there was a significant interaction for subjective concentration levels between office design and study phase (*F*_1,65_ = 8.59, *p* =.005). This significant interaction could not be replicated (*F*_2,158_ =.93, *p* =.40) (92). However, further model analyses revealed cross-lagged effects for disengagement on concentration between T2 and T3 (*β* = −.19, *p* =.030), i.e. between two weeks after the enrichment with plants and three and a half months later. Additionally, the cross-lagged effects for concentration on disengagement were significant between T2 and T3 (*β* = −.16, *p* =.040). While green vs. lean condition had no direct effect on concentration, it had an indirect effect on concentration at T3 mediated through disengagement at T2. Hence, the green office condition reduced call center agents’ disengagement, which consequently had a positive effect on their concentration. The office design had no effect on objective productivity measures (i.e. total time in min call center agents spend on the phone) (92).

In Nieuwenhuis et al. (92), consultants in the green office space perceived their *subjective productivity* to be greater after the introduction of plants (*F*_1,57_ = 3.81, *p* =.056, 95% *CI* [−.01,.16]) compared to consultants in the lean office space indicating a decrease in subjective productivity (*F*_1,57_ = 3.04, *p* =.086, 95% *CI* [−.51,.03]). Yet, both simple effects failed to reach the critical significance level. In Nieuwenhuis et al. (92), cognitive performance tasks, representing office-based tasks (i.e. information management, processing, and vigilance) were used as opposed to subjective questionnaires. Consultants who completed the *vigilance* task in the green office condition, outperformed their counterparts in terms of time taken to complete it (*F*_1,30_ = 7.91, *p* =.009). No significant effect of office design was found for the other tasks. In Sianoja et al. (95), a main effect on the within-person level for green exercise (*β* =.36, SE =.12, *p* < .01) on afternoon concentration was found. Hence, green exercise during lunchtime significantly predicted better afternoon concentration. Moreover, an indirect effect of green exercise *via* lunchtime enjoyment on afternoon concentration was identified (*ab* =.07, 95% *CI* [.02,.13], *p* < .05); with a proportion of the mediated effect of.16 (*CI* [.04,.42], *p* < .05). In Calogiuri et al. (93) fatigue was excluded from analyses because the normality assumption was not satisfied.

#### Recovery and Restoration

##### Risk of Bias

Three out of four study outcomes on recovery and restoration showed a high overall risk of bias as a result of contamination between groups and poor adherence (*deviations from intended intervention*; Brown_3). DeBloom_a_1 and _b_1 displayed a higher overall risk due to the same reasons as mentioned above (see cognitive ability). Only one study outcome was assessed as raising *some concerns* (Calogiuri_3).

##### Study Outcomes

No significant effects of green exercise were found on either HR or HRV in response to stress nor recovery from stress (97). In de Bloom et al. (94) the effect sizes for recovery experiences (i.e. relaxation, detachment) and enjoyment after lunchtime green exercise remained trivial (*d* < 0.15) during the intervention period. After the intervention period the green exercise group showed lower levels of enjoyment of their lunch breaks (*d* = 0.38). Contrarily, the within-group effects in de Bloom et al. (94) on relaxation (*d* =.66, *p* < .05), detachment (*d* =.61, *p* < .05) and enjoyment (*d* =.47, *p* < .05) were considerably higher and significant compared to baseline and control-group during the intervention period. Postintervention effects remained trivial again, ranging from *d* = −.12 to.09. A small (*d* =.23) but nonsignificant effect was reported for lunchtime restoration postintervention. Only trivial or no effects were found for lunchtime restoration during the intervention period (*d* =.17; control group: *d* =.47), evening restoration during the intervention period (*d* = −.03) and evening restoration postintervention (*d* =.12) (94). In the same study in fall (94) green exercise participants indicated higher levels of restoration after the lunch break (*d* =.33), and in the evening (*d* =. 26) during the intervention period. No effects were found postintervention. Statistically significant differences were reported between green exercise and indoor exercise groups on perceived restorativeness for both exercise sessions: Green exercise environment scored higher on fascination (*p* < .01) and being away (*p* < .01, respectively *p* =.01 for the first exercise session) (93).

#### Work and Life Satisfaction

##### Risk of Bias

All five study outcomes in the category work and life satisfaction displayed a high overall risk of bias. This was repeatedly due to *deviations from intended interventions* (Bang_3, Nieuwenhuis_a_1, Nieuwenhuis_b_1) and *poor measurement of the outcome* (Bang_3, DeBloom_a_3, DeBloom_b_3). Especially for study outcomes in Bang et al. (89) employees may have felt unlucky to have been assigned to the control group (i.e. no attempt of blinding) and therefore sought the experimental intervention (i.e. engagement in green exercise). Moreover, there were inconsistencies in the description and usage of the measurement tool, which favored the experimental group (Bang_3).

##### Study Outcomes

The green exercise group reported significantly higher quality of life than the control group postintervention (*p* =.020) (89). In de Bloom et al. (94), no effects of green exercise on job satisfaction were found. Whereas in de Bloom et al. (94), a small effect (*d* =.22) was reported for job satisfaction during the intervention period compared to baseline. The effect did not persist for postintervention (*d* =.08). Nieuwenhuis et al. (92) reported that the workplace satisfaction of consultants increased from baseline to postintervention (*F*_1,65_ = 23.0, *p* < .001), but, importantly, the effect was not qualified by office design (*p* =.23). Thus, workplace satisfaction increased in both conditions. However, in Nieuwenhuis et al. (92) call center agents in the green office condition showed a significant increase in their workplace satisfaction (*F*_1,79_ = 22.18, *p* < .001) two weeks after plants were introduced (T2). Moreover, the follow-up measure T3 (i.e. three and a half month after plants were introduced) showed that workplace satisfaction had only slightly changed in the long term (*F*_1,79_ = 2.10, *p* =.151). The office design resulted in a significant direct effect on disengagement (*β* = −.15, *p* =.040) and workplace satisfaction (β =.37, *p* < .001) at T2. Thus, the call center agents in the green office space were less disengaged and more satisfied with their workspace. Further model analyses showed that disengagement predicted workplace satisfaction (baseline to T2, *β* =.19, *p* =.025; T2 to T3, *β* = −.24, *p* =.019). Hence, disengagement served as a moderator between office design and workplace satisfaction. Working in a green office reduced employees’ disengagement and in turn fostered workplace satisfaction.

#### Psychophysiological Indicators of Health

##### Risk of Bias

Two out of three study outcomes displayed a high overall risk of bias judgment because of contamination between intervention and control, respectively comparison group (*deviations from intended intervention*; Bang_1, Brown_1) and very poor adherence (Brown_1). Furthermore, employees in the NBI condition were actively encouraged to be more active and to engage in additional green exercise within the experimental time frame, leading to a) additional health-related behaviors that differed between groups and b) to *some concerns* in the *measurement of the outcome* subjective physical activity (Bang_1).

##### Study Outcomes

No statistically significant effect of green exercise was found for anthropometric measurements (waist circumference, body weight, BMI) (89, 97), nor for body composition parameters (muscle mass, bone muscle mass, body fat), or bone density (89).

Concerning the cardiovascular parameters, no significant differences in CVD risk score, resting HR, or HRV were identified (97), while mixed findings were observed for *BP*: Bang et al. (89) reported no significant difference in either diastolic or systolic BP. Brown et al. (97) found a significant group*time effect in favor of green exercise for systolic BP (*F* = 5.53, *p* < 0.01), but not diastolic BP. Whereas Calogiuri et al. (93) found a marginally significant between-groups effect for diastolic BP (*F* = 4.91, *p* = 0.05), but not systolic BP. A significant between-groups effect was found for cortisol-awakening response with respect to increment (CAR_i_) (*F* = 4.56, *p* = 0.04) but not for cortisol-awakening response with respect to ground (CAR_G_), nor for cortisol morning serum concentration (93). Furthermore, a significant between-groups effect in favor of green exercise was found for self-reported weekly physical activity (89, 93), future exercise intention (*B* = 1.79, *p* < 0.01), and biking in nature (*B* = 0.84, *p* = 0.04) (93). No significant effects were reported for self-reported biking indoors [(93), see (91)]. Differently, no significant effect was found for objectively measured lunch-time physical activity or aerobic fitness (97).

## Discussion

The present study intended to (*i)* provide a synthesis of current quantitative research that applied NBIs within an occupational setting, and (*ii)* evaluate their effectiveness on employees’ mental health and well-being outcomes. The 10 studies included displayed a large degree of heterogeneity in terms of design, nature exposure, assessed outcomes, and measurement tools, which precluded the possibility to conduct a meta-analysis. The wide range of applied outcomes was categorized into clustered dependent variables: (*i)* mental health indices, (*ii)* cognitive ability, (*iii)* recovery and restoration, (*iv)* work and life satisfaction, and (*v)* psychophysiological indicators of health. Furthermore, the different types of NBIs were grouped into three different types of NBIs: green exercise (five studies), nature savoring (two studies), and green office space (three studies).

### Principal Findings

The findings of this review offer support for the positive impact of NBIs on employees, especially in relation to mental health indices. Five out of six studies found in fact statistically significant positive effects of the respective NBIs on self-rated mental health indices (90, 93, 95, 97, 98). The effects of NBIs in the workplaces the other clustered outcome variables (cognitive ability, recovery and restoration, work and life satisfaction, and psychophysiological indicators) were less consistent. Cognitive ability, which was investigated by the majority of studies [excluding (89, 97, 98)], showed only small to medium effects. The evidence on recovery and restoration, which was assessed in only four studies, was ambivalent, with two studies demonstrating positive effects (93, 94) and two studies stating no significant effects (94, 97). Of the five studies investigating the effects of NBI on work and life satisfaction, two were unable to demonstrate effects (92, 94), one found only a marginal effect (94), and two found statistically significant effects (89, 92). For what concerns the psychophysiological indicators of health, for which information was available only for three studies, it should be noted that each study included different anthropometric, hormonal, and/or cardiovascular measurements. Among these, only three measurements in two different studies showed statistically significant effects (93, 97).

With respect to the type of NBI, nature savoring was the only NBI that demonstrated exclusively significant findings (90, 98). Contrary to nature savoring, green exercise and green office space studies reported positive associations, but also nonsignificant and mixed findings. However, it cannot be deduced from these findings whether visual exposure to nature (i.e. green office space) combined with the mindful appreciation of natural elements (i.e. nature savoring) or physical activity in nature (i.e. green exercise) is more advantageous for the mental health and well-being of employees. On the other hand, outcome assessments of nature savoring studies displayed lower risk of bias (all scored “only” *some concerns* in the overall risk-of-bias judgment). All green exercise studies and green office space interventions, with one exception each (92, and 93, respectively), were on the other hand deemed to be of *high overall risk-of-bias*.

### Weaknesses of Evidence

The included studies conducted NBIs in real-world environments, contributing to high ecological validity. However, weaknesses and shortcomings have been identified in terms of scope and description of natural environment, methodological quality, lack of study of confounding and mediating variables, impact the interpretability of results, and grade of evidence.

A challenge in this field is that the natural environment includes many diverse types, characteristics and amounts of green and blue spaces (e.g., wilderness areas vs. urban parks). All of the included studies were administered in green space. Based on the authors account, only Largo-Wight et al. (98) might have included presence of blue space. However, based on the available data, it is impossible to determine whether (or to what extent) the employees were actually exposed to environments including views of water, as the employees were merely instructed to take a work break outdoors in nature. Evidence suggests that the mental health benefits resulting from nature exposure varies not only by characteristics and quality of green space (106), but is also influenced by the proportion of blue space available (107). All green exercise and nature savoring studies lacked an adequate description of the natural environment, except for Matsunaga et al. (90) and Calogiuri et al. (93) that captured the greenery *via* photography. One reason for the lack of description can be attributed to the fact that the NBIs were carried out in multiple natural settings within one trial [e.g., (94, 98)]. However, measurement tools assessing and describing the quantity and quality of authentic natural spaces are well established [e.g., (13)].

The reviewed studies presents a number of methodical limitations, and are thus subjected to bias and confounding, displaying *some concerns* or an estimated *high* in the overall risk-of-bias assessment.

In some studies, the choice of the instruments used to assess mental health indices is, in our opinion, questionable. For instance, BDI is strictly speaking an instrument for evaluating the severity of depression symptoms [e.g., (108)]. In research and practice it is often used as a screening instrument, contrary to the field of application recommended by the authors (109). Furthermore, mental illness is no longer simply understood as the opposite end of the spectrum to mental health, but instead as part of a two continua model (56). Only two studies (93, 98) stated the reliability (Cronbach’s alpha) of the measurements used. Particularly for applied one item measurements (see **Table 3**), a minimum of test-retest reliability should have been reported. However, single-item measures may be adequate and suffice for some one-dimensional psychological constructs (e.g., job satisfaction) (110) to further reduce the response burden and length of questionnaires (111).

A main weakness concerns the lack of follow-up measurements and therefore, studies only describes short-term results, with the exception of Calogiuri et al. (93) and Nieuwenhuis et al. (92). Furthermore, the sample sizes and group sizes were generally small (mean of samples: n = 28) with absence of an adequate sample size calculation (excluding 89) to approve the number of included employees. Yet, this is pivotal in intervention studies (112). It is therefore ambiguous whether no significant effects or small effects were a consequence of insufficient power or a true indication on the outcomes measured. Particularly, de Bloom et al. (94) reported small effect sizes without reaching the statistical significance level. Other limitations concern the omission of sufficient description of baseline characteristics. Thus, sociodemographic group comparability was rarely explicitly described and analyzed.

Another major limitation is represented by insufficient treatment of confounding variables at an individual level as well as within the context. The concept of nature connectedness as individuals’ affective, cognitive, and experiential aspects of human-nature relationship has emerged as a correlate of psychological well-being (113, 114). Calogiuri et al. (93) was the only study that assessed nature connectedness at baseline—even though a meta-analysis suggests nature connectedness is rather a mediator of the benefits to good mental health (e.g., greater positive affect) (115). All green exercise and nature savoring studies stated the months in which the interventions took place. However, it is important to acknowledge the climatic differences both across and within countries, thus the information about months is not sufficient. Four out of six studies (90, 93, 94) provided additional information such as weather conditions and temperatures, which are a critical success factor in interventions taking place outdoors (17).

There are further numerous confounding or mediating variables that were not or could not be controlled within trials: environmental stressors (e.g., poor air quality), additional green exercise in leisure time (e.g., participants in 90 were encouraged to do so), work-related variables (e.g., exhaustion), physical spillover of green office design (e.g., 92), and social interaction. Particularly in green exercise studies in which employees could choose to walk individually or with co-workers (94, 97), a social component might have had an additional pleasant benefit. In Largo-Wight et al. (98), the participants were instructed to sit outside and focus on natural elements. However, it is unclear, whether participants actually sat still or engaged in some sort of green exercise at the same time (e.g., walking to a bench). An issue that is prevalent in occupational research is the over-representation of female participants (116, 117) This was the case for six studies, whereas one showed an over-representation of males conducted in the financial sector (97) and three others were more or less balanced for gender (92, 93). Lack of blinding assessors, lack of blinding of participants to hypotheses, and lack of preregistrations was also an issue in many of the studies included.

Finally, another common limitation includes the absence of information about adverse events, side effects, unintended consequences, and safety issues. Poor reporting of adverse events was also acknowledged by the CERT (i.e. standardized method for reporting exercise programs), for which all green exercise studies scored rather low. There was a general absence of a preregistered protocol prior to study commencement (only 97, had preliminary registered the protocol). Hence, there might be potential risk of bias of due to inadequate analysis or selective reporting.

### Strengths and Limitations of Review

To the best of the authors’ knowledge, this is the first systematic review to address workplace NBIs both on actual employees and in real workplace settings. A main strength of this review is the identified transdisciplinary theoretical framework as a valuable foundation to guide the synthesis process and clarify the expected outcomes (118). Furthermore, multiple databases were used including Medline that independently provides a satisfying recall (approx. 90%) when searching for high quality studies within occupational health (119). The nonrestriction of publications in languages other than English resulted in two articles from the East Asian region. This was particularly important due to the practice of “Shinrin-yoku” (taking in the forest atmosphere or forest bathing), a traditional Japanese practice and growing parallel development within the East Asian region (120). To reduce the degree of subjectivity in this review, two independent reviewers with the help of a third researcher conducted the screening, the eligibility assessment, the data extraction and the risk-of-bias assessment. Moreover, a standardized tool to evaluate the risk of bias in the findings of included studies was used (86).

The main limitations and reasons for the weakness of evidence in this review were the paucity and heterogeneity of available studies. The outcome variables varied substantially among studies, e.g., for the outcome variable mental health indices seven different questionnaires were used: BDI, SF-8, PAAS, PSQ, STAI, POMS and one single-item instrument measuring perceived stress/strain. This made it impossible for us to conduct a quantitative synthesis of the findings (meta-analysis), which would have allowed to pool results together and estimate effect overall sizes.

While this study categorized each implemented NBI in one of three NBI types (green exercise, nature savoring, green office space), there were substantial divergences in delivery, activities, length and intensity. Moreover, it might be that additional NBIs studies may exist but escaped the search criteria, despite thorough exploration of appropriate keywords prior to final search. Due to the complex and broad vocabulary used for NBIs (55), there is the risk of having overlooked significant key words. Another limitation refers to the dispensation of database alerts after November 2018 – thought more hits do not necessarily mean more high quality studies. For an optimal trade-off between sensitivity and specificity of the search, information specialists and librarians could have been supportive as the recall of search is dependent on the skills of the end-user (119).

Within this systematic review, the new Cochrane RoB tool (RoB 2) was used and the assessment was conducted and implemented with the use of its manual and the recommend Excel tool. However, it was the first time that the authors used this particular tool. Disagreements between RoB judgments occurred, in particular because of terminology in studies being used inconsistently and because the interventions were complex [e.g., (121)]. Moreover, algorithm malfunctions within the Excel tool were identified (e.g., signaling questions indicated high risk of bias, algorithm resulted in low).

### Implications for Practice

The knowledge base for formulating clear practical recommendations limits one to only tentative suggestions due to the scarcity of studies. The implementation of green exercise and nature savoring requires commitment and personal initiatives. Participation in the planning of the intervention design predicts higher commitment during the intervention period, and in turn positively influences intervention outcomes (122). This mechanism is particularly key for conducting NBIs, given that the experience of control is an important feature for a successful recovery process (123).

In general, the application of NBIs in terms of green exercise and nature savoring appears to be independent of the work sector, size of the company, and financial resources. This unique attribute differentiates NBIs from other work health promotion efforts. Moreover, to date no specific contraindications or negative side effects of NBIs have been determined (32). However, serious illness or allergies (e.g., hay fever) that prevent employees from going outside need to be determined prior to implementation.

### Recommendations for Future Research

Research within occupational health psychology must constantly balance the tension between internal and external validity (124). Thus, interventions may have a lack of effect either as a result of weaknesses in their design or failures in implementation (125). Due to the abundant room for further progress in determining the effectiveness of NBIs within workplaces, only some will be highlighted. Firstly, there is a need for clearer definition and classifications of NBIs, and for an increased detail in reporting of them. Accurate descriptions, for example of the natural environment, might be beneficial to obtain more fine-grained analyses of different intervention modalities. With the high variability inherent in NBIs in work settings to date, it is crucial to clarify in future studies what natural environments with what natural stimuli and what activities are beneficial. A lack of transparency impedes reproducibility and generalizability of results, and consequently the accumulation of robust evidence.

When conducting workplace intervention research, blinding of employees from group allocation and conditions is often challenging or not feasible, leading to contamination of the control group. Cluster randomization may ameliorate this shortcoming (126). Furthermore, future studies require valid and reliable outcome measures combining physiological correlates of mental health and questionnaire-based outcome parameters. For the latter, research should consider measuring outcomes tailored to the work context and measuring constructs that derive from the theoretical framework applied, for example, workplace flourishing [Flourishing-at-Work Scale, FAWS; (57)], recovery experiences [Recovery Experiences Questionnaire; (41)], perceived restorativeness [Restorative Components Scale, RCS; (127)]. In particular, a consensus on what should be measured prior, during and following NBIs is reasonably needed in order to make research comparative. Thus, future studies should investigate not only short-term effects of NBIs, but also intermediate and long-term effects, especially regarding acquired resilience (128) and to establish how implementation holds up to a cost-benefit analysis over long term (124). Measurements of work productivity might also provide important information to evaluate the cost-effectiveness of NBI in the workplace.

Recent conclusions indicate a conceptual overlap and similarities between the environmental psychology concept of restoration and recovery in occupational health (129), calling for an amalgamation of both concepts from work, organizational, and environmental psychology (94). More research needs to be undertaken, to investigate this interesting prospect and consideration of contemporary conceptual frameworks (73, 130) would help generate additional testable hypotheses. It may also be possible in subsequent reviews, with the proliferation of theory-driven research, that theories could potentially be used to cluster the empirical articles, which would enable meaningful comparisons of the different explanations of diverse NBI’s.

Future research needs to take various individual (e.g., age, nature connectedness) and workplace factors (e.g., organizational culture) into account to explore whether some employees or organizations might benefit more or less than others. This will give further insights into whether NBIs should be conducted on the organizational-level (i.e. targeting large groups of employees) as currently done or on the individual-level (e.g., only targeting employees that score high or low on nature connectedness or languishing). Another interesting field is to investigate effects of technological nature (131) for two reasons: firstly, to eliminate confounding variables that appear in authentic nature and, secondly, to include employees that might have reduced or no access to direct nature, for example, in industrial and manufacturing sites.

Despite suggested future research opportunities, NBIs in the workplace present complex interventions and therefore challenges denoting a high degree of connectivity between components and a large number and variability of outcomes (132). The Medical Research Council outlines best practice for design, implementation and evaluation of complex interventions. In particular, prior to developing an intervention, a thorough theoretical understanding is required to anticipate the changes and causal chain induced by the intervention. Similarly, Hartig et al. (42) emphasize a need for theory to advise research which nature types, and which features of those types, are relatively effective for particular outcomes. Process evaluation has a key role in any stage of the intervention, to assess the feasibility, optimize its design, evaluate effectiveness, transferability and generalizability (133). In particular, process evaluation is needed to reveal implementation failures as limited effects may be a result of implementation issues rather than genuine ineffectiveness.

## Conclusions

This systematic review adds to our understanding of how NBIs can contribute to employees’ mental health and well-being. The results showed predominantly positive effects on mental health indices and cognitive ability, but mixed findings for recovery and restoration as well as for psychophysiological indicators of health and life and work satisfaction. From the paucity and heterogeneity of studies, it is apparent that experimental research of NBIs in actual workplaces is in its infancy. This research area is challenging and complex, which resulted in high overall risk-of-bias of the individual studies. There is especially a need for theory-driven and well-designed trials.

## Author Contributions

SG designed and led the overall study, conducted the literature search, screened the potentially eligible papers, conducted and reviewed the data extraction, acted as a third part in resolving disagreements in the risk-of-bias assessment, and drafted the manuscript. TM contributed substantially in the ideation of the study and in determining the final eligibility of potentially eligible papers. JB-B provided substantial contribution in conducting the literature search, screening of eligible papers, and extracting the data from the selected papers. DD and GC conducted the risk-of-bias assessment. All authors substantially contributed to the writing up of various manuscript stages leading to the final version, which was approved by all authors.

## Funding

The authors’ participation in this research was entirely funded by their respective institutions. The study did not receive any specific grant from funding agencies in the public, commercial, or not-for-profit sectors. The first author received funding support from the Department of Physical Education and Sport Sciences at the University of Limerick (PESS internship award) and Enterprise Ireland during the research phase. Economical support for Open Access publication was received from INN University.

## Conflict of Interest

The authors declare that the research was conducted in the absence of any commercial or financial relationships that could be construed as a potential conflict of interest.
